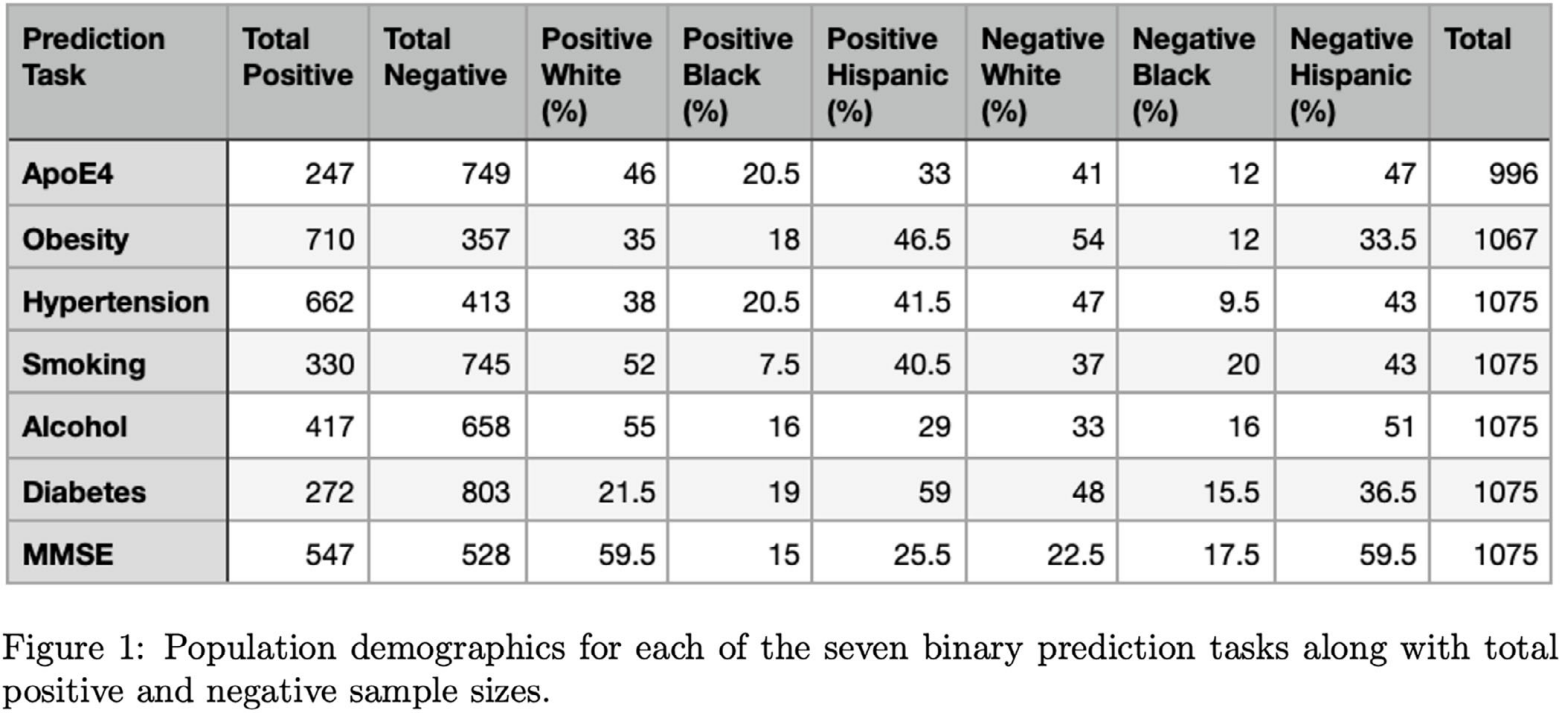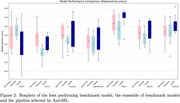# Automated and Unbiased Machine Learning Predictions of Alzheimer's Risk Factors in an Ethnically Diverse Dataset using Cortical Thicknesses

**DOI:** 10.1002/alz70856_100888

**Published:** 2025-12-25

**Authors:** Douglas M. J. Wyllie, Maitrei Kohli, Robert Leech, Philip SJ Weston, James H. Cole

**Affiliations:** ^1^ UCL Hawkes Institute, University College London, London, United Kingdom; ^2^ UCL Institute of Health Informatics, University College London, London, United Kingdom; ^3^ Institute of Psychiatry, Psychology and Neuroscience, King's College London, London, United Kingdom; ^4^ Dementia Research Centre, UCL Queen Square Institute of Neurology, London, United Kingdom

## Abstract

**Background:**

The 2024 Lancet Commission identified 14 major risk factors contributing to 45% of dementia cases globally. Many relate to brain structure changes, potentially mediating brain health on the pathway to dementia. This study explores these factors using brain imaging measures in binary prediction tasks.

The ethnically diverse Health and Aging Brain Study‐Health Disparities (HABS‐HD) dataset was used to reduce bias and improve model generalisability, a known issue in medical AI.

AutoML, a machine learning framework, reduces experimenter bias by automating pipeline selection in a data‐driven manner.

**Method:**

The HABS‐HD study includes over 3,000 Black, Hispanic, and White individuals with clinical data and MRI brain scans. Six risk factors were investigated‐ApoE ε4 allele presence, hypertension, obesity, smoking, diabetes, alcohol consumption‐plus MMSE as a cognitive marker. A summary of population demographics for each task can be seen in Figure 1.

In the AutoML framework a multiverse of all possible pipelines is constructed. Bayesian optimisation is then used in a lower‐dimensional representation of this space to select the optimal pipeline for each task.

Nine standard benchmark machine learning models plus an ensemble of all benchmark models were compared to the AutoML method. The mean cortical thicknesses of 68 brain regions based on the Desikan‐Killiany atlas provided by HABS‐HD were used as features. Task‐specific cutoffs included a BMI of ≥ 27.5 for obesity, MMSE ≥ 29, and a history of daily smoking. Each model was evaluated using stratified K‐Fold (3 folds, 3 repeats).

**Result:**

In 5 of 7 prediction tasks AutoML showed improved performance over the best benchmark model and ensemble. AutoML achieved a balanced accuracy of 57.9% in the classification of MMSE, compared to 56.7% and 55.5% for the best benchmark model and ensemble. Results for all can be seen in Figure 2.

**Conclusion:**

In the challenging task of predicting risk factors exclusively from cortical thickness values, AutoML demonstrated its ability to adapt to each task. Despite identical input data, AutoML selected unique pipelines for 5 out of 7 tasks, highlighting the lack of a universal model and showing the potential value of AutoML in Alzheimer's research.